# *Saccharomyces cerevisiae* for lignocellulosic ethanol production: a look at key attributes and genome shuffling

**DOI:** 10.3389/fbioe.2024.1466644

**Published:** 2024-09-25

**Authors:** Kindu Nibret Tsegaye, Marew Alemnew, Nega Berhane

**Affiliations:** ^1^ Department of Biology, Gondar College of Teachers Education, Gondar, Ethiopia; ^2^ Institute of Biotechnology, University of Gondar, Gondar, Ethiopia

**Keywords:** bioethanol, genome shuffling, lignocellulose, *Saccharomyces cerevisiae*, strain improvement

## Abstract

These days, bioethanol research is looking at using non-edible plant materials, called lignocellulosic feedstocks, because they are cheap, plentiful, and renewable. However, these materials are complex and require pretreatment to release fermentable sugars. *Saccharomyces cerevisiae*, the industrial workhorse for bioethanol production, thrives in sugary environments and can handle high levels of ethanol. However, during lignocellulose fermentation, *S. cerevisiae* faces challenges like high sugar and ethanol concentrations, elevated temperatures, and even some toxic substances present in the pretreated feedstocks. Also, *S. cerevisiae* struggles to efficiently convert all the sugars (hexose and pentose) present in lignocellulosic hydrolysates. That’s why scientists are exploring the natural variations within *Saccharomyces* strains and even figuring out ways to improve them. This review highlights why *Saccharomyces cerevisiae* remains a crucial player for large-scale bioethanol production from lignocellulose and discusses the potential of genome shuffling to create even more efficient yeast strains.

## 1 Introduction

The growing energy crisis and rising greenhouse gas concerns are driving the development of bioethanol, a transportation fuel derived from renewable sources. Bioethanol is already a significant contributor to renewable energy in many countries, even being the leading bio-based product globally in terms of volume and economic value (Favaro et al., 2019). However, current bioethanol production often relies on food crops as feedstock. Earlier reports showed that sugarcane and sugar beet account for about 40% of the global bioethanol production, with starch-based feedstocks contributing the remaining 60% (Dhar et al., 2012). This raises concerns about potential competition with food production and indirect land-use change emissions. Additionally, the cost of production can be high, with feedstock costs accounting for up to 70% of the final price (Topaloglu et al., 2023). This highlights the importance of developing bioethanol production processes that utilize readily available and inexpensive resources. In Europe, some pilot and demonstration bioethanol plants from lignocellulosic biomass are either operational or in the process of being commissioned (https://www.etipbioenergy.eu/fact-sheets/ethanol-fact-sheet, accessed on 27 August 2024).

At the global scale, bioethanol is primarily produced from either starch or selected edible agricultural crops like corn, sugar cane, and sugar beet (Busic et al., 2018; Tse et al., 2021). The utilization of agricultural residues and other low-value carbohydrate sources for bioethanol production is of great interest due to economic and environmental factors (Favaro et al., 2019). Lignocellulosic materials, including residues from agriculture and forestry, are gaining prominence in bioethanol studies owing to their abundance, renewable nature, and sustainable characteristics. Furthermore, these materials do not pose a competition with food or fuel crops, rendering them a favorable choice (Tse et al., 2021; Arora et al., 2015; Zhang and Geng, 2012). Lignocellulosic biomass primarily comprises cellulose (40%–60%), hemicellulose (20%–40%), and lignin (10%–25%) (Deng et al., 2023). The breakdown of lignocellulosic biomass through hydrolysis produces hexose sugars like glucose, predominantly from cellulose, and pentose sugars such as xylose and arabinose from hemicellulose (Cunha et al., 2019; Nijland and Driessen, 2020). Therefore, an ideal microorganism should have the capability to ferment both hexose and pentose sugars to enhance the economic viability of bioethanol industry.

Despite the considerable potential it holds, the utilization of lignocellulosic substrates in the production of bioethanol is faced with numerous obstacles. These challenges include the necessity of a pre-treatment phase, inhibitors that arise from the pre-treatment process, and the incapacity of *S. cerevisiae* to simultaneously ferment glucose and xylose (Deng et al., 2023; Sandri et al., 2023). Industrial strains for lignocellulosic ethanol production need to be ethanol tolerant, temperature-tolerant (typically above 40°C), osmo- and pH-tolerant, and inhibitor-tolerant (Jansen et al., 2017). Therefore, developing an *S. cerevisiae* strain with high capacity for stress tolerance and co-fermentation of glucose and xylose has been the subject of extensive research in recent years.

Efficient utilization of lignocellulosic biomass for biofuel production requires a proficient microorganism capable of fermenting the feedstocks while tackling the challenges associated with pretreatment and hydrolysis techniques. The target for the industry should be to achieve a minimum of 90% theoretical yields, which equates to around 0.511 g of ethanol per g of glucose consumed (Della-Bianca et al., 2013; Walker and Walker, 2018), where the residual sugar is <2 g/L (Tao et al., 2012). *S. cerevisiae* emerges as the predominant microorganism utilized in industrial ethanol manufacturing due to its remarkable fermentation capabilities and ability to withstand stress factors (Topaloglu et al., 2023; Wang et al., 2022). In the realm of industrial-scale ethanol production, *S. cerevisiae* consistently attains a yield exceeding 90% of the theoretical maximum (Della-Bianca et al., 2013). Hence, even a marginal enhancement in ethanol yield could result in hundreds of millions of dollars in extra profits annually (Ruchala et al., 2019).

However, *S. cerevisiae* encounters various environmental stressors throughout the commercial fermentation process. For example, optimal saccharification typically occurs at temperatures ranging from 45°C to 50°C, implying that *S. cerevisiae* must withstand elevated temperatures and high ethanol concentrations during the latter stages of the fermentation process. Furthermore, strains intended for very high-gravity fermentation (>250 g/L glucose) must exhibit tolerance to high sugar concentrations (Wang et al., 2022). This review highlights the industrially relevant attributes of *S. cerevisiae* and the contribution of genome shuffling in achieving these traits.

## 2 Desired attributes of strains to ferment lignocellulosic biomass

Lignocellulosic biomass represents a vast, renewable resource for biofuel production. The conversion of this biomass into fermentable sugars and subsequently into bioethanol or other valuable chemicals is a promising alternative to fossil fuels. However, the efficient fermentation of lignocellulosic hydrolysates requires robust yeast strains with specific attributes to overcome the challenges posed by the complex nature of the biomass. The essential characteristics of an industrial strain necessary for fermenting lignocellulosic hydrolysate include rapid fermentation kinetics, high ethanol yield, tolerance to ethanol, sugars, and fermentation inhibitors, and efficient utilization of both hexoses and pentose sugars (Zhang and Geng, 2012; Mukherjee et al., 2014; Zhao et al., 2024). However, the conditions encountered during bioethanol fermentation present several challenges, including exceedingly high levels of sugar and ethanol, elevated temperatures, and the presence of toxic compounds. Consequently, the development of an industrial yeast strain with enhanced thermotolerance, osmotolerance, and resistance to secondary metabolites has become imperative for bioethanol production (Wawro, 2018; Kong et al., 2018). Exploring the natural diversity within *Saccharomyces* is widely perceived as an intriguing approach to discover superior bioethanol strains and gain insights into the challenges faced by yeast cells during bioethanol production (Mukherjee et al., 2014).

Although several bacteria, such as *Zymomonas mobilis* and genetically modified *Escherichia coli*, can ferment sugars, *S. cerevisiae* remains the preferred organism for industrial ethanol production (Dien et al., 2003; Moyses et al., 2016; Techaparin et al., 2017). This preference primarily stems from its high tolerance to ethanol and its ability to ferment under strictly anaerobic conditions. Additionally, *S. cerevisiae* is resilient to low pH and resistant to bacteriophage infection, all of which are crucial factors in large-scale industrial processes (Moyses et al., 2016; Techaparin et al., 2017). Therefore, a strong multiple stress-tolerance is the desirable characteristic for *S. cerevisiae* when different feedstocks are used for economical industrial ethanol production. This review examines the desired attributes of yeast strains for fermenting lignocellulosic biomass, focusing on their ability to tolerate elevated temperature and high ethanol concentration, efficiently utilize available sugars, tolerate inhibitory compounds, and perform under very high-gravity fermentation conditions.

### 2.1 Temperature tolerance

Numerous critical factors must be considered in the production of industrial ethanol. One pivotal environmental factor affecting ethanol production is temperature. The optimal temperature range for yeast growth is typically between 25°C and 35°C. However, heat stress significantly impacts industrial ethanol production (Techaparin et al., 2017). According to Prado et al. (2020), ethanol-producing strains in Brazil ferment within the range of 28°C–33°C, with mills employing water coolers to regulate temperature. Nevertheless, addressing the challenge of ethanol production under high temperatures can be achieved through the utilization of thermotolerant microbes (Prado et al., 2020; Phong et al., 2022). Hence, it is generally imperative to explore the natural diversity of *Saccharomyces* strains to find superior thermotolerant bioethanol strains from new and less explored areas.

Interest in producing ethanol at high temperatures has been driven by several advantages, including enhanced metabolism, accelerated fermentation rates and yield, decreased media viscosity, reduced energy consumption, and minimized contamination risks (Techaparin et al., 2017; Prado et al., 2020; Phong et al., 2022; Pattanakittivorakul et al., 2019). Moreover, the predominant method for producing lignocellulosic ethanol involves separate hydrolysis and fermentation processes, which increase costs and require distinct vessels for saccharification (40°C–50°C) and fermentation (30°C–35°C) (Yuan et al., 2017; Choudhary et al., 2017). Thus, there is a need to develop a simultaneous saccharification and fermentation process where the average temperature for both steps is approximately 40°C which will allow simultaneous saccharification and fermentation of lignocellulosic biomass (Prado et al., 2020). Mostly, mesophilic yeasts are employed for fermentation; however, a simultaneous saccharification and fermentation approach necessitates thermotolerant yeast capable of withstanding the optimal temperature range of saccharifying enzymes. Consequently, a critical aspect of bioethanol production via simultaneous saccharification and fermentation is the isolation of thermotolerant ethanologenic yeasts. Abrams and Brem (2022) showed that *S. cerevisiae* maintained viability at temperature 37°C, whereas *S. paradoxus* became inviable. Novel thermotolerant *S. cerevisiae* strains obtained by different researchers had been reviewed and presented in Table 1.

**TABLE 1:** A list of thermotolerant *S. cerevisiae* strains that can produce ethanol from different lignocellulosic substrates.

*Strains*	Sources	Opt T°C	Additional information	EtOH yield	Residual sugar	Ref.
*S. cerevisiae* DMKU 3-S087	Fresh and rotten fruits, palm sugar, honey, soil and water in sugar factories, fermented fruits, and traditional alcoholic beverages	40°C	- Molasses used as substrate − 200 g/L total fermentable sugars	0.36 g/g (70.45% theoretical efficiency)	NR	Pattanakittivorakul et al. (2019)
*S. cerevisiae* KKU-VN8	Fruits, flowers and other sources, such as banana, papaya, grape, orange, apple, mango, Vietnamese apple flowers, longan flowers, papaya flowers, alcoholic beverages, soil and sawdust	Up to 45°C	- Sweet sorghum juice - Ethanol tolerance 13% v/v − 200 g/L total sugar	89.32 g/L at 40°C (96.32% theoretical efficiency)	NR	Techaparin et al. (2017)
*S. cerevisiae HG1.1*	Soil sample	40°C	- Pineapple waste hydrolysate	0.48 g/g (93.61% theoretical efficiency	21.79 g/L	Phong et al. (2022)
*S. cerevisiae* TBRC 12151	Commercial and culture collections	40°C	- Cassava starch - Ethanol tolerance 12%	64.9 g/L (77.7% theoretical efficiency)	NR	Kruasuwan et al. (2023)
*S. cerevisiae* TC-5	Culture collections	40°C	- Corncob residue via fed- batch SSF	0.478 g/g glucose (93.5% theoretical yield)	NR	Boonchuay et al. (2021)
*S. cerevisiae JRC6*	Rotten fruit samples and distillery waste samples	40°C	- Paddy straw hydrolysate	0.5 g/L (97.8% theoretical yield	0.2 ± 0.27	Choudhary et al. (2017)
*S. cerevisiae* UV-20	Slightly rotted pears	37°C	- Tolerant to 50% glucose and 21% of ethanol	121.18 g/L	NR	Yi et al. (2018)

^a^ Simultaneous saccharification and hydrolysis; opt, optimum; EtOH, ethanol; NR, Not reported.

Numerous researchers have identified many thermotolerant strains of *S. cerevisiae* from various sources, demonstrating their ability to produce ethanol at elevated temperatures (Table 1). In nearly all cases, the optimal temperature for ethanol production was reported to be 40°C. Notably, *S. cerevisiae* KKU-VN8, *S. cerevisiae* JRC6, *S. cerevisiae* TC-5 and *S. cerevisiae* HG1.1 achieve over 90% of the theoretical ethanol yield at 40°C, meeting the industrial threshold. On the other hand, the residual sugar in many of the strains was not reported though the industrial requirement is less than 2 g/L. Some of the strains like DMKU 3-S087 and TBRC 12151, though thermotolerant, produced ethanol below the industrially significant threshold of 90% theoretical yield. Besides thermotolerance, some strains, such as *S. cerevisiae* UV-20, tolerate 50% glucose and exhibit remarkable ethanol tolerance of up to 21% (v/v) (Yi et al., 2018). In contrast, *S. cerevisiae* KKU-VN8 and *S. cerevisiae* TBRC 12151 resist 13% (v/v) and 12% (v/v) ethanol, respectively (Table 1).

Several studies have been conducted to improve the thermotolerance of *S. cerevisiae* through different mechanisms. For example, Yang et al. (2023) modified *S. cerevisiae*’s sterol composition using CRISPR–Cas9 system to boost thermotolerance. Others used adaptive laboratory evolution (Zhang B. et al., 2023), regulating expression of *spt23* (Lu et al., 2022), regulation of transcription factors (Gan et al., 2021), manipulation of heat shock protein genes (Zhang M. L. et al., 2023) to enhance yeasts’ response to temperature. Since thermotolerance is just one aspect, it is essential to evaluate additional characteristics of a strain beyond its thermotolerance. Hence genome shuffling can be a powerful tool because it has the potential to improve multiple traits simultaneously by creating a combinatorial effect from beneficial mutations across the genome.

### 2.2 Ethanol tolerance

*Saccharomyces cerevisiae* can metabolize sugar to produce ethanol at levels that are harmful to most organisms (Morard et al., 2019; Riles and Fay, 2019). However, there are variations in ethanol tolerance among different strains (Morard et al., 2019; Vamvakas and Kapolos, 2020). During the final stages of bioethanol production, *S. cerevisiae* strains are exposed to high ethanol concentrations. This ethanol stress often slows down or halts fermentation, resulting in reduced ethanol yield (Snoek et al., 2015; Tao et al., 2012). Therefore, new strains with improved ethanol tolerance are needed to increase efficiency and maximize ethanol production at an industrial scale.

The ethanol tolerance of *S. cerevisiae* is a complex trait controlled by numerous genes and cannot be easily modified by altering a single gene. Research has shown that improvements in ethanol tolerance result from extensive genomic changes (Tao et al., 2012). Nevertheless, there existed disparities in ethanol tolerance across various yeast strains (Morard et al., 2019; Vamvakas and Kapolos, 2020; Wolf et al., 2023). The severity of a given ethanol concentration can vary between strains. For example, if one strain is tolerant to 5% ethanol and another to 10%, only the first strain will activate stress-specific pathways when both are exposed to 5% ethanol. This makes it challenging to compare their ability to cope with ethanol stress since 5% ethanol is severe only for the first strain (Wolf et al., 2023).

Different researchers employed various strategies to enhance the ethanol tolerance of *S. cerevisiae*. Morard et al. (2019) identified that polysomy of chromosome III plays a significant role in ethanol tolerance. Strains with an extra copy of this chromosome showed higher ethanol tolerance, which could be reversed by restoring euploidy. Others have shown that the overexpression of certain genes can enhance ethanol tolerance. For instance, Varize et al. (2022) reported that the overexpression of the *TRP1* gene (involved in tryptophan biosynthesis) or MSN2 gene (general stress response activator) increased ethanol tolerance up to 14%. Wang et al. (2022) identified genes like *ASP3* (encodes L-asparaginase II), *ENA5* (encodes a P-type ATPase to reduce cytotoxicity), *YOL162W* and *YOR012W* of unknown function, and two transcription factors (*Crz1p*, *Tos8p-*that may regulate multiple-tolerant phenotypes) could be used as gene targets to improve multiple stress-tolerance (high ethanol, high temperature, high glucose, high salt) and ethanol production capacities of *S. cerevisiae*.

Similarly, Samakkarn et al. (2021) reported a transcription factor called *Znf1* that plays a central role in ethanol stress response by activating genes for glycerol and fatty acid production to preserve plasma membrane integrity. Comparative genomics has revealed that ethanol-tolerant strains exhibit distinct genetic modifications, where the ethanol tolerance strains are associated with a combination of beneficial alleles across the genome (Wohlbach et al., 2014). To cope with ethanol stress, strains alter the levels of key metabolites, including amino acids, organic acids, and fatty acids, indicating a reprogramming of metabolic fluxes (Ming et al., 2019). In their findings, Haas et al. (2019) suggested that ethanol tolerance is under natural evolutionary fitness-selection for an optimum phenotype that would tend to eliminate alleles of large effect. Despite substantial research endeavors in recent years dedicated to pinpointing evolutionary pathways that could boost ethanol tolerance, this trait remains partially elucidated (Morard et al., 2019; Ming et al., 2019).

Ethanol tolerance is also influenced by temperature. Studies by Riles and Fay (2019) identified that amino acid polymorphisms in the *SEC24* gene contribute to ethanol sensitivity at elevated temperatures. Other researchers, like Techaparin et al. (2017) and Kruasuwan et al. (2023), obtained strains that could tolerate up to 12% ethanol at 40°C and up to 13% ethanol at 45°C, respectively. However, there are limited studies reporting on the interplay between thermal and ethanol stress responses.

Natural isolates of *S. cerevisiae* from various environments exhibit inherent ethanol tolerance. For instance, strains isolated from vineyards in the Western Cape, South Africa displayed high phenotypic diversity including the ability to grow at 45°C and in the presence of 20% (v/v) ethanol (Jansen et al., 2017). It has been found by Zheng and Wang (2015) that isolates from human-associated environments exhibited higher stress tolerance compared to those from forests unaffected by human activity. This may be attributed to evolutionary adaptations to environments influenced by human activities. Dhar et al. (2012) demonstrated that yeasts exhibit adaptive responses to fluctuating stress conditions, characterized by the development of cross-protection and anticipatory gene regulation. Thus, investigating the genetic diversity of such kind of isolates can provide valuable insights into the mechanisms of ethanol tolerance (Minnaar and Haan, 2023).

Therefore, ethanol tolerance in *S. cerevisiae* is a multifaceted trait governed by genetic, physiological, and environmental factors. Future research should explore the complex interplay between these factors to develop robust yeast strains capable of sustaining high ethanol yields in industrial bioethanol production.

### 2.3 Tolerance to lignocellulosic-derived inhibitors

Lignocellulosic biomass is composed of cellulose, hemicellulose, and lignin, which are tightly bound together. Pretreatment is necessary to break down this complex structure to enhance the enzymatic hydrolysis of cellulose into fermentable sugars. Common pretreatment methods include acid hydrolysis, steam explosion, and alkaline treatment. Although these methods are effective in breaking down the biomass, they produce various inhibitory byproducts such as acetic acid, formic acid, furfural, 5-hydroxymethylfurfural (HMF), and phenolic compounds (Li et al., 2022; Divate et al., 2022). These inhibitors can negatively impact the growth, metabolism, and fermentation performance of *S. cerevisiae*, reducing ethanol yield and overall productivity (Cunha et al., 2019; Li et al., 2022; Ko et al., 2019; Brandt et al., 2019). Therefore, understanding yeast responses and adaptations to these stresses is crucial for developing strategies to improve yeast resilience and bioconversion efficiency from lignocellulosic biomass (Cunha et al., 2019).

Enhancing yeast robustness against lignocellulosic-derived inhibitors is crucial for transitioning to a bio-based economy (Moreno et al., 2022; Wang et al., 2015). Engineering yeast to overexpress genes involved in the detoxification of inhibitors has shown to improve tolerance. For example, overexpression of *ARI1* (encodes aldehyde reductase) appears to confer higher tolerance to aldehyde inhibitors, thereby increasing the growth rate and ethanol production capacity of *S. cerevisiae* in an aldehyde-containing environment (Divate et al., 2022). Wang et al. (2015) also demonstrated that overexpressing the key genes *PRO1* or *INO1*, which enhance the synthesis of proline or myo-inositol, significantly increases yeast tolerance to furfural, acetic acid, and phenol (FAP). Ren et al. (2024) demonstrated the effectiveness of a combined X-ray radiation and adaptive evolution approach in creating furfural-resistant yeast mutants. Another strategy involves combining beneficial traits from multiple strains through iterative cycles of DNA recombination and selection, known as genome shuffling, resulting in strains with enhanced tolerance to inhibitors (Cunha et al., 2019).

Natural *S. cerevisiae* isolates exhibit niche-specific phenotypic and metabolic diversity that has evolved to overcome external stresses, utilize available resources, and thrive in challenging environments (Jansen et al., 2017; Witt et al., 2019). In contrast, industrial and laboratory strains lack these adaptations due to domestication (De Witt et al., 2019). For example, Jansen et al. (2017) identified strains from various vineyards with high tolerance to ethanol (20% v/v), temperature (45°C), pH (2–11), and lignocellulosic inhibitor cocktails (25%). Other studies suggest that a strain’s exposure to specific environments influences its phenotypic resistance to lignocellulosic inhibitors (Narayanan et al., 2017). Pre-cultivating yeast in lignocellulosic hydrolysate containing furfural and HMF induces the expression of NADPH-dependent oxidoreductases, which convert aldehyde groups into less inhibitory furfuryl alcohols. Consequently, pre-exposing cell populations to moderate levels of inhibitors during the pre-cultivation phase can enhance fermentation efficiency of lignocellulosic substrates (Narayanan et al., 2017).

Therefore, natural strains can be a valuable resource for mitigating engineering constraints by studying the molecular mechanisms involved in phenotypic variance and informing future industrial strain improvements for lignocellulosic hydrolysates (Witt et al., 2019). While significant progress has been made through genetic engineering and the use of natural isolates, further research is needed to fully understand the complex interactions between yeast cells and inhibitors.

### 2.4 Co-fermentation of hexose and pentose sugars

The utilization of all types of sugars obtained from the process of biomass pretreatment, namely hexoses and pentoses, plays a crucial role in the advancement of viable processes within the framework of a bio-refinery. Microorganisms have the ability to convert hexoses into various end products through biochemical pathways. However, a significant obstacle lies in the effective conversion of pentoses, especially when they are present alongside hexoses. The enhancement of sugar conversion during co-fermentation poses a substantial challenge that researchers and industry professionals are actively working to address (Sandri et al., 2023; Jamaluddin et al., 2023). In industrial bioethanol production, *S. cerevisiae* is commonly utilized due to its rapid digestion of hexose sugars and high tolerance to ethanol compared to other species. However, wild *S. cerevisiae* strains are unable to ferment pentose sugars, specifically xylose, despite it being the second most abundant sugar in lignocellulosic hydrolysates (Zhang and Geng, 2012; Li et al., 2024; Zhu et al., 2020). In contrast, non-*Saccharomyces* yeasts such as *Kluyveromyces marxianus*, *Scheffersomyces* (*Pichia*) *stipitis*, *Pachysolen tannophilus*, and *Candida shehatae* are capable of fermenting pentose sugars. Nevertheless, they have not been considered for large-scale production due to their low ethanol yield. Consequently, the majority of research on pentose fermentation in yeast has focused on genetically engineering *S. cerevisiae* strains to incorporate heterologous xylose metabolic pathways (Cunha et al., 2019; Moyses et al., 2016; Ko et al., 2016; Liu et al., 2018; Nijland et al., 2023).

Efforts to genetically modify *S. cerevisiae* to incorporate xylose metabolic pathways have had limited success and does not enable yeast to rapidly utilize xylose. Several limitations still need to be addressed, including glucose inhibition and slow xylose transport, cofactor imbalance in the xylose reductase/xylitol dehydrogenase pathway, functional expression of a heterologous xylose isomerase, the low efficiency of downstream pathways and low ethanol production (Hou et al., 2017; Sharma et al., 2021; Ko et al., 2016). To facilitate the conversion of D-xylose into bioethanol, two distinct pathways have been integrated and optimized in *S. cerevisiae*: the xylose reductase/xylitol dehydrogenase (XR/XDH) pathway and the xylose isomerase (*XI*) pathway (Cunha et al., 2019; Ko et al., 2016; Liu et al., 2018; Nijland et al., 2023). Regardless of the approach employed in genetic engineering, *S. cerevisiae* strains designed to metabolize D-xylose rely on the activity of xylulose kinase (*Xks1*), which catalyzes the conversion of D-xylulose to D-xylulose-5-phosphate at the cost of one ATP molecule (Nijland et al., 2023). Given that this enzymatic reaction represents a crucial step in D-xylose metabolism, it is conceivable that precise control of *XKS1* expression may be necessary. Nevertheless, the regulatory mechanisms associated with D-xylose metabolism in *S. cerevisiae* seem to be absent, possibly due to the fact that this particular sugar is not recognized as a primary carbon source (Sharma et al., 2021; Han et al., 2010). Through combined efforts of reinforcing the pathway of xylose catabolism and elevating the fermentation temperature, Tran et al. (2020) achieved a simultaneous co-fermentation of lignocellulosic hydrolysates, composed of 39.6 g/L glucose and 23.1 g/L xylose, within 24 h yielding 0.48 g/g ethanol (94% theoretical maximum).

Enhancing *S. cerevisiae’s* ability to efficiently utilize pentose sugars can also be achieved by engineering endogenous hexose transporters (*Hxt*) to function as pentose transporters. However, these modified transport proteins remain susceptible to glucose-level controlled degradation (Nijland and Driessen, 2020; Nijland et al., 2016). A more successful approach involves engineering the endogenous *Hxt* transporter family and using evolutionary selection for D-glucose insensitive growth on pentose sugars. This strategy has led to the identification of a critical and conserved asparagine residue in *Hxt* transporters that, when mutated, reduces D-glucose affinity while maintaining D-xylose affinity (Nijland and Driessen, 2020).

While several xylose-consuming *S. cerevisiae* strains have been constructed by the heterologous expression of the *XR/XDH* or the *XI* pathways, the reported ethanol yields from xylose are still far below the theoretical yield (Hou et al., 2017; Ko et al., 2016; Hoang et al., 2018). On the other hand, there is a lack of consensus in the few studies comparing the use of the different xylose-consuming pathways: while all describe the strains containing the *XI* pathway as capable of reaching higher ethanol yields than the ones containing the *XR/XDH* pathway, the *XI* role in xylose consumption rates and ethanol productivity is not clear (Karhumaa et al., 2007; Kobayashi et al., 2018; Li et al., 2016).

At industrially relevant concentrations, the co-consumption of D-xylose and D-glucose has been realized through transporter engineering when expressed in a strain lacking hxt1-7 (hexose transporters) and gal2 (galactose permease) genes (Nijland and Driessen, 2020). Although this is the main achievement, to translate this method to real industrial conditions will still be a challenge because sugar metabolism flux is distributed over multiple sugars, as the maximal capacity of primary metabolism is exploited. Specifically, D-xylose utilization and growth rates are still significantly lower as compared to D-glucose, and thus a careful balance is necessary throughout fermentation (Nijland and Driessen, 2020). Therefore, in addition to the directed genetic manipulations, random mutational approaches would provide better insights into complex network linked with xylose metabolism. This would assist in the development of an improved strain with mixed sugar utilization/fermentation potential (Sharma et al., 2021).

One of the random mutational approaches to develop an effective strain with improved capability is protoplast fusion (Sharma et al., 2021). It causes random mutations in the genomes of microorganisms without requiring any pre-targeted approach. This is applicable for developing inter-specific, intraspecific, and inter-generic, intra-generic super hybrids with higher capability. It is a significant tool for genetic manipulation as it resolves the barrier to genetic exchange imposed by conventional mating systems. It is particularly useful for industrially important microorganisms (Zhang and Geng, 2012; Sharma et al., 2021; Kahar and Tanaka, 2014).

Several researchers have employed genome shuffling to produce fusants/hybrids with desired traits. For instance, Pasha et al. (2007) constructed a fusant strain, F11, showing a 0.459 g/L ethanol yield with 90% fermentation efficiency. Likewise, Sharma et al. (2021) employed protoplast fusion to derive a hybrid strain from *S. cerevisiae* LN and *Pichia stipitis* NCIM 3498. They showed higher ethanol tolerance (10%), enhanced *XR*, *XDH*, and *XKS* activities, and exhibited the ability to ferment glucose in lignocellulosic hydrolysate, with xylose consumption (∼40%) surpassing that of *S. cerevisiae* LN. Zhang and Geng (2012) obtained a potential recombinant yeast strain, after two rounds of genome shuffling (ScF2), from *S. cerevisiae* and *P. stipites* that can utilize a high concentration of xylose (100–250 g/L xylose). This hybrid, ScF2, produced ethanol more rapidly than the naturally occurring xylose-fermenting *P. stipitis*, with an improved ethanol titer and a much greater increase in xylose tolerance. In another study, the gene shuffling protocol using the highly homologues hexose transporters family provides a powerful tool to enhance the D-xylose affinity of *Hxt* transporters in *S. cerevisiae* to increase the D-xylose uptake flux at low D-xylose concentrations (Nijland et al., 2018). Through protoplast fusion and metabolic engineering, Zhao et al. (2024) created a recombinant *S. cerevisiae* strain, BLH510, capable of co-fermenting glucose, xylose, cellobiose, and xylooligosaccharides while exhibiting tolerance to inhibitors commonly present in lignocellulosic hydrolysates (109). These findings suggest that *S. cerevisiae* strains can be genetically modified to efficiently utilize a wider range of sugars found in lignocellulosic biomass.

### 2.5 High- and very-high-gravity fermentation

Ethanol production continues to advance within industrial settings. One notable innovation that has significantly transformed industrial ethanol production is very high-gravity (VHG) fermentation, also known as high-sugar-concentration fermentation (Camargos et al., 2020; Cruz et al., 2020). Generally, sugar concentrations for ethanol production can be divided into normal gravity (<180 g/L total sugars), high gravity (180 - 240 g/L of total sugars), and very high gravity (≥250 g/L of total sugars) (Bai et al., 2008; Deesuth et al., 2015). Both HG and VHG technologies have the potential to reduce distillation costs and yield up to 120 g/L ethanol (Cruz et al., 2020; Liu et al., 2012) and facilitate increased ethanol production without necessitating plant capacity or workforce adjustments. Very high gravity fermentation can achieve more than 15% (v/v) of ethanol, compared to the average of 10%–12% (v/v) that is observed in most distilleries (Puligundla et al., 2011). VHG technology, in particular, holds promise as a highly effective industrial ethanol production method due to its ability to generate high ethanol yields, minimal waste, and cost-effectiveness (Tse et al., 2021; Cruz et al., 2020; Arshad et al., 2017). Compared to submerged and solid-state fermentation, very high gravity fermentation has the greatest potential for high ethanol yields (Tse et al., 2021). However, under VHG fermentation conditions, yeasts undergo multiple stresses due to increased metals and sodium ions, nutrient stresses, increased temperatures, acidic conditions, osmotic stress, and increased ethanol concentrations (Tse et al., 2021; Varize et al., 2022; Puligundla et al., 2011).

Several studies reported ethanol production under HG and VHG fermentation conditions. For instance, Camargos et al. (2020) reported an ethanol concentration of 104.4 g/L from 222 g/L total reducing sugars under fed-batch fermentation conditions with a maximum ethanol productivity of 2.98 g/L.h from 209 g/L total reducing sugars. In a similar study, an ethanol concentration of 135 g/L, with ethanol productivity of 4.42 g/L.h, and a 90% ethanol yield were achieved at a sugar concentration of 300 g/L (Cruz et al., 2020). These findings demonstrate the feasibility of HG and VHG fermentation for commercial-scale ethanol production. However, VHG fermentation is not without drawbacks, including unfermented sugars and the risk of stuck fermentation, leading to prolonged fermentation times (Tao et al., 2012). Osmotic stress, glycerol production, and substrate and ethanol inhibition are among the adverse effects associated with VHG fermentation (Tao et al., 2012; Camargos et al., 2020). Tao et al. (2012) used a combination of metabolic engineering (to delete glycerol gene) and genome shuffling to circumvent the limitations of VHG fermentation and improved the bioethanol production performance of *S. cerevisiae*. They found that the fusant, SZ3-1, was capable of effectively fermenting 280 g/L glucose within 72 h while simultaneously maintaining a high fermentation rate, enhanced ethanol tolerance, and a low glycerol yield. Similarly, Zheng et al. (2013) obtained isolates with significantly enhanced stress tolerance and ethanol titer under VHG conditions after whole-genome shuffling of the *S. cerevisiae* ZTW1. They attributed the stress tolerance largely due to copy number variations in large DNA regions.

Some studies claimed that it is difficult to change properties under the control of multiple genes through classical breeding, metabolic engineering, and other genetic manipulation methods with specific genes or pathways as targets (Teixeira et al., 2012; Wang and Hou, 2010). However, whole genome engineering approaches involving yeast cell ploidy manipulation and genome shuffling can be employed to improve the fermentation performances under VHG conditions (Teixeira et al., 2012; Hou et al., 2015). Hou et al. (2015) compared the methods of metabolic pathway modification, cell ploidy manipulation, global transcription machinery engineering, and genome shuffling to improve ethanol production under VHG fermentation. The results suggested that genome shuffling was the most effective way to significantly manipulate yeast strains where by ethanol production was enhanced by up to 11% more than the control, reaching 120 g ethanol/L in 35 h using 300 g glucose/L. The results demonstrated that genome shuffling was valuable for creating yeast strains with desired multiplex traits during VHG fermentations. However, further studies are necessary to verify this finding.

## 3 Potential of genome shuffling in microbial strain improvement

Microbial strains have been extensively utilized for the production of various valuable commodities across different industries. However, the majority of natural strains cannot be directly used for industrial purposes due to limitations in their productivity, stability, and resistance to environmental stresses. To address these challenges, one effective approach is strain improvement (Chen et al., 2020; Magocha et al., 2018; Sanghavi et al., 2020). Among the techniques available for strain improvement, genome shuffling has emerged as a promising method for rapid enhancement of microbial strains, yielding new strains by combining whole genomes of multiple parental microorganisms using recursive protoplast fusion principles (Chen et al., 2020; Magocha et al., 2018; Hospet et al., 2023). It is a laboratory evolution method involving the combinatorial evolution of complex phenotypes in whole organisms by genome-scale and recursive recombination of mutants (Biot-Pelletier and Martin, 2014). Genome shuffling has brought a major breakthrough in the strain-improvement concept as it is found to be effective and reliable for expressing complex phenotypes (Magocha et al., 2018).

Genome shuffling has some attributes similar to those of classical strain-improvement as both offer genomic diversification and screening for improved strains. In case of random mutagenesis, it generates microbial strains with desired traits and does not necessitate knowledge of the microbe’s genetic makeup, however, it is a labor-intensive process. In contrast, recombinant DNA technology, metabolic engineering, and genome editing techniques are rational genetic engineering methods with a narrow scope of application, as they require a comprehensive understanding of the genetics of the target strain and specialized genetic tools (Chen et al., 2020). To accelerate the enhancement of complex traits and swiftly observe phenotypic improvements in industrially significant strains, genome shuffling has emerged as a more practical and innovative whole-genome improvement technique. Genome shuffling facilitates recombination among multiple parents in each generation, and through repeated fusion of protoplasts, fusants successfully amalgamate genetic traits from diverse parents (Hospet et al., 2023). In conventional protoplast fusion between 2 cells with diverse genetic backgrounds, robust recombinants exhibiting traits from both parents can emerge. Here, the fusion of only two parents in a single generation induces recombination. Conversely, genome shuffling facilitates multiple rounds of genome fusion and the amalgamation of numerous parents from each generation (Zhang and Geng, 2012; Chen et al., 2020; Hospet et al., 2023) without requiring genome sequencing data (Magocha et al., 2018; Biot-Pelletier and Martin, 2014). It can be used in most laboratories without expensive equipment (Gong et al., 2009). Furthermore, genome shuffling allows for the fusion of isolated DNA fragments with a cell’s genome, merging genomes of organisms spanning distant taxonomic groups (Luna-Flores et al., 2016). This approach significantly enhances the likelihood of obtaining a high yield of the strain and unquestionably expands the genetic diversity of the offspring (Wawro, 2018).

## 4 Application of genome shuffling on *Saccharomyces cerevisiae* to enhance bioethanol production

Research shows that genome shuffling enhances the phenotypic traits of *S. cerevisiae* (Table 2). This technique is widely used to improve the desirable performance of microbes, particularly for complex traits that are challenging to achieve through traditional genetic engineering methods (Gong et al., 2009). Chen et al. (2020) summarized the application of genome shuffling in increasing the yield of various microbial metabolites, enhancing strain tolerance, and improving substrate utilization across different microbial strains. This review specifically focuses on applying genome shuffling to improve *S. cerevisiae* strains for bioethanol production.

**TABLE 2:** Genome-shuffled *S. cerevisiae* strains and their desired traits for bioethanol production.

Parental strains	GS strategy	Goal and achievement	Ref.
Industrial diploid *S. cerevisiae* strains (Sun049 and Sun224)	UV mutagenesis and Spore mating	Xylose fermentation under temperature and acid co-stress - 33.1 g/L at 38°C with 20 mM acetic acid and 15 mM formic acid, ethanol yield - 0.33 g/g xylose (65% Theoretical efficiency)	Inokuma et al. (2017)
Thermotolerant and ethanol tolerant industrial *S. cerevisiae* CE25 mutant	DES mutagenesis and Protoplast fusion	Improved ethanol production by 4.1 times at 42°C, 7.9 times at 0.55% (v/v) acetic acid, and 8.9 times at 0.2% (v/v) furfural at 40°C	Lu et al. (2012)
*S. cerevisiae* and *P. stipites*	Electroporation	Ethanol titer of 51 g/L from 150 g/L initial xylose, ethanol titer decreased at high xylose concentration and in the pressure of glucose and xylose mixture	Zhang and Geng (2012)
*Spathaspora passalidarum* NRRL Y-27907 and *S. cerevisiae* ScY01	Genome shuffling/intergeneric followed by adaptive evolution	Shuffled strain showed a 1.39-fold increase in xylose consumption and 2-fold increase in EtOH productivity at 40°C than parents, Evolved strain exhibited more efficient xylose consumption and ethanol accumulation rates than shuffled strains. In glucose and xylose mixture, both shuffled and evolved strains depleted xylose in less time than parents but deplete glucose at lower rate than *S. cerevisiae*	Lin et al. (2019)
Industrial *S. cerevisiae* strain KF-7	ARTP mutagenesis, Sporulation and hybridization	Hybrid grow better at 10 %–14% (v/v) ethanol, at 43°C and 45°C, under 350- 450 g/L glucose, 1- 2 mol/L NaCl, and 1- 3 mol/L sorbitol Ethanol production 10.14% - 81.02% higher than the parent strains in stress conditions	Wang et al. (2021)
Thermotolerant and high ethanol producing *S. cerevisiae* strains	Spore-to-cell hybridization	Hybrids give an ethanol yield of 0.47 g/g glucose at 41°C and pH 3.5 (91.2% efficiency)	Benjaphokee et al. (2012)
*S. cerevisiae* strains, Z8 and Z15	Sporulation and hybridization	Shuffled strain yield 3.11%, 10.31%, and 10.55% higher ethanol than the parent under regular, increased heat, and HG fermentation conditions	Zheng et al. (2011)
*S. cerevisiae* W5 and XR, XDH, and XK genes	Recombinant strains Protoplast fusion	Ethanol yield of hybrid (0.4 g/g glucose and xylose) 17.65% higher than the yield by parent strains	Jingping et al. (2012)
*S. cerevisiae* and *P. stipites*	Protoplast fusion	Xylose utilization - 34%, ethanol yield - 0.447 g/g, ethanol tolerance - 15% (v/v)	Jetti et al. (2018)
*S. cerevisiae* ZTW1	Sporulation and hybridization	Hybrids with enhanced stress tolerance (ethanol, heat, and H_2_O_2_) and ethanol titer under VHG conditions obtained Ethanol production 3%–11% more than the parent strains under VHG A hybrid showed less than 2% residual sugar (1.95 ± 0.20) under VHG conditions after 75 h fermentation meeting industrial standard	Zheng et al. (2013)
*S. cerevisiae* SM-3	UV mutagenesis and Protoplast fusion	Improved ethanol productivity (95%–100%) of the theoretical maximum at 45°C–48°C from 20% (w/v) glucose and tolerate 25% (v/v) ethanol	Shi et al. (2009)
*S. cerevisiae*	*GPD2* gene knockout, and sporulation and hybridization	Increased ethanol yield by 8% under VHG conditions than the parent strain and enhanced ethanol tolerance	Tao et al. (2012)
*S. cerevisiae* strain FY2	Adaptive laboratory evolution, and mating and zygote-pulling	A hybrid exhibited a 100% fitness increase to 0.9 g HMF/L, another strain exhibited a 13% fitness increase to 0.7 g/L furfural	Cheng et al. (2015)
*S. cerevisiae* strain W303	EMS mutation and sexual and asexual reproduction	Increased ethanol yield by up to 13% and resistance to ethanol at 15% (v/v)	Hou (2009)
Ethanol Red	Expression cassette with 13 genes, EMS mutagenesis, sporulation and evolutionary engineering	Hybrid produced 32% more ethanol than the parent in simultaneous saccharification and fermentation Ethanol yield of up to 0.48 g/g was obtained from 40 g/L D-xylose (94% of the theoretical maximum)	Demeke et al. (2013)
Industrial *S. cerevisiae* TH-AADY strain	Sexual and asexual reproduction	Ethanol yield of a strain constructed by genome shuffling enhanced by up to 11% more than the control within 35 h using 300 g glucose/L	Hou et al. (2015)
*C. tropicalis* KBKTI.10.5.1 and *S. cerevisiae* DBY1	Electroporation	Ethanol produced by mutants on acid hydrolysate increased by 14.94%–26.58% from parent strains but only by 1.35% on alkaline hydrolysate	Jamaluddin et al. (2023)
*S. cerevisiae* LN and *P. stipitis* NCIM 3498	Protoplast fusion	Fusants showed higher tolerance to 10% ethanol and enhanced *XR*, *XDH*, and *XKS* activities. However, low ethanol yield (0.17- 0.24 g/g on 2% xylose)	Sharma et al. (2021)
*S. cerevisiae* 2013	EMS mutagenesis and protoplast fusion	Hybrids are able to grow at 42°C and 18% ethanol and yielding 10.83% higher ethanol than parental strain, and 15.16% over the industrial strain *S. cerevisiae* HBY3	Orosco et al. (2017)

Keys: ARTP, atmospheric and room temperature plasma; GS, genome shuffling; NR, not reported; EtOH, ethanol; VHG, very high gravity.

Shuffled strains of *S. cerevisiae* exhibited enhanced traits such as xylose utilization, temperature resilience, ethanol production, and high-gravity fermentation (Table 2). For example, Jetti et al. (2018) developed a fusant strain, *S. cerevisiae* SP2-18, capable of utilizing 34% xylose and tolerating 15% (v/v) ethanol after two rounds of genome shuffling. Utilizing super-strains with elevated temperature, acidity, and ethanol tolerance has proven economically viable for ethanol production. Benjaphokee et al. (2012) created fusants from thermotolerant and high ethanol-producing *S. cerevisiae* strains, resulting in strains that could tolerate 41°C and a pH of 3.5, producing an ethanol yield of 0.47 g with 91.2% theoretical efficiency. Similarly, Orosco et al. (2017) obtained a shuffled strain capable of growing at 42°C and in 18% ethanol, yielding 10.83% higher ethanol than parental strain and 15.16% over the industrial strain *S. cerevisiae* HBY3.

**FIGURE 1:**
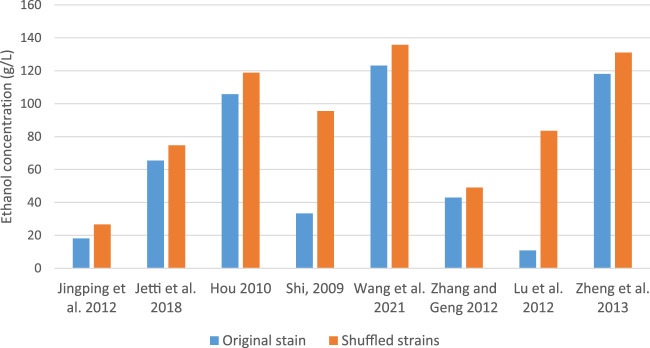
Comparative bioethanol production by parent and shuffled *S. cerevisiae* strains.

Others like Inokuma et al. (2017) developed a xylose-utilizing *S. cerevisiae* strain with improved fermentation ability under heat and acid co-stress using genome shuffling. The shuffled strain successfully inherited desirable traits from parental strains and exhibited high xylose fermentation ability under heat and acid co-stress, attributed to the upregulation of genes in Hyb-8 associated with cellular transition metal ion homeostasis. Lu et al. (2012) suggested that trehalose accumulation in recombinant strains contributes to thermotolerance and other stress tolerances. Lin et al. (2019) also created hybrid strains with improved glucose-xylose co-fermentation properties at high temperatures by combining genome shuffling with adaptive evolution. Hou et al. (2015) compared the ethanol production of strains generated by four methods: metabolic pathway modification, cell ploidy manipulation, global transcription machinery engineering and genome shuffling. Notably, ethanol yield in a strain created by genome shuffling enhanced by 11% as compared to the parent strain. As illustrated in Figure 1, shuffled *S. cerevisiae* strains consistently produced higher ethanol yields compared to their respective parental strains. The more pronounced differences observed in the studies of Shi et al. (2009) and Lu et al. (2012) may be attributed to variations in the specific strains used and the fermentation conditions employed.

Overall, genome shuffling has been employed to confer traits such as thermotolerance, ethanol tolerance, xylose utilization, high-gravity fermentation, and multi-stress tolerance. Studies have demonstrated that just two or three rounds of genome shuffling can generate potential *S. cerevisiae* hybrids with desirable characteristics. Therefore, employing genome shuffling for strain enhancement serves as a crucial method to address complex phenotypic features and facilitate the rapid evolution of strains with industrial significance.

## 5 Conclusion and future prospects

Bioethanol derived from lignocellulosic feedstocks presents a compelling option for the development of sustainable biofuels. *Saccharomyces cerevisiae* stands out as the outstanding microorganism for industrial bioethanol production due to its exceptional fermentation capabilities and tolerance to harsh environments. However, the commercial fermentation process exposes *S. cerevisiae* to a multitude of stresses. For optimal lignocellulosic bioethanol production, industrial yeast strains require a specific range of traits, including rapid fermentation rates, high ethanol yields, tolerance to fermentation inhibitors and various sugars, and efficient utilization of both hexose and pentose sugars. Unfortunately, most naturally occurring strains lack the requisite productivity or stress tolerance for direct industrial application.

Strain improvement strategies present a promising avenue to overcome these challenges. Wild isolates of *S. cerevisiae* harbor an extensive reservoir of genetic diversity that can be tapped through genetic modifications to explore the genetic foundations of novel phenotypes. Genome shuffling has emerged as a potent tool for rapidly enhancing complex traits critical to industrial applications. This technique facilitates precise recombination among multiple parental strains in each generation. By conducting repeated protoplast fusions, these “fusants” effectively consolidate the genetic strengths inherited from numerous parents. However, genome shuffling alone may not suffice to achieve strains meeting industrial requirements and should therefore be complemented with other genetic manipulation strategies.

Overall, the combination of lignocellulosic feedstocks, *S. cerevisiae*, and strain improvement via genome shuffling holds immense promise for the future of sustainable bioethanol production. This approach can potentially yield optimized yeast strains that enhance the efficiency of bioethanol conversion from lignocellulose, thereby advancing the environmental sustainability and economic competitiveness of the biofuel industry. Moreover, future research should consider reporting the theoretical efficiency and residual sugar activity of microbial strains before asserting their industrial applicability.
